# Engineered Neural Tissue (EngNT) Containing Human iPSC-Derived Schwann Cell Precursors Promotes Axon Growth in a Rat Model of Peripheral Nerve Injury

**DOI:** 10.3390/bioengineering12090904

**Published:** 2025-08-23

**Authors:** Rebecca A. Powell, Emily A. Atkinson, Poppy O. Smith, Rickie Patani, Parmjit S. Jat, Owein Guillemot-Legris, James B. Phillips

**Affiliations:** 1 UCL Centre for Nerve Engineering, Department of Pharmacology, UCL School of Pharmacy, University College London, London WC1N 1AX, UK; rebecca.powell1293@gmail.com (R.A.P.); emily.atkinson@ucl.ac.uk (E.A.A.); poppy.smith.20@ucl.ac.uk (P.O.S.); o.guillemot-legris@ucl.ac.uk (O.G.-L.); 2 The Francis Crick Institute, London NW1 1AT, UK; rickie.patani@ucl.ac.uk; 3 Department of Neuromuscular Diseases, Queen Square Institute of Neurology, University College London, London WC1N 3BG, UK; 4 MRC Prion Unit at UCL, UCL Institute of Prion Diseases, University College London, London W1W 7FF, UK; p.jat@prion.ucl.ac.uk

**Keywords:** tissue engineering, human induced pluripotent stem cells (hiPSCs), Schwann cells, Schwann cell precursors, nerve injury, nerve repair

## Abstract

Tissue engineering has the potential to overcome the limitations of using autografts in nerve gap repair, using cellular biomaterials to bridge the gap and support neuronal regeneration. Various types of therapeutic cells could be considered for use in aligned collagen-based engineered neural tissue (EngNT), including Schwann cells and their precursors, which can be derived from human induced pluripotent stem cells (hiPSCs). Using Schwann cell precursors may have practical advantages over mature Schwann cells as they expand readily in vitro and involve a shorter differentiation period. However, the performance of each cell type needs to be tested in EngNT. By adapting established protocols, hiPSCs were differentiated into Schwann cell precursors and Schwann cells, with distinctive molecular profiles confirmed using immunocytochemistry and RT-qPCR. For the first time, both cell types were incorporated into EngNT using gel aspiration–ejection, a technique used to align and simultaneously stabilise the cellular hydrogels. Both types of cellular constructs supported and guided aligned neurite outgrowth from adult rat dorsal root ganglion neurons in vitro. Initial experiments in a rat model of nerve gap injury demonstrated the extent to which the engrafted cells survived after 2 weeks and indicated that both types of hiPSC-derived cells supported the infiltration of host neurons, Schwann cells and endothelial cells. In summary, we show that human Schwann cell precursors promote infiltrating endogenous axons in a model of peripheral nerve injury to a greater degree than their terminally differentiated Schwann cell counterparts.

## 1. Introduction

Peripheral nerve injury has an immediate, debilitating impact on millions of people every year, resulting in loss of function as well as potential long-term neuropathic pain and disability. For a severe injury, where there is a large gap in the tissue, the current clinical treatment is the nerve autograft. However, the autograft is restricted by its limited availability, donor site morbidity and possible size mismatch [1]. Nerve autografts provide a supportive environment of aligned Schwann cells and extracellular matrix to guide nerve regeneration. Engineered neural tissue (EngNT) has the potential to reconstruct this natural environment with its aligned collagen matrix containing columns of elongated therapeutic cells, therefore offering a potential alternative to the autograft [2].

The regenerative properties of the peripheral nervous system are heavily dependent on the supportive response of Schwann cells to axonal damage [3]. After an injury, Schwann cells are reprogrammed, altering their phenotype and expression profile to support nerve repair. The resulting repair Schwann cells form tracks, called bands of Büngner, in the nerve tissue distal to the injury. The bands of Büngner guide regenerating axons and prevent misdirection of reinnervation [3,4]. The role and benefit of Schwann cell-based therapies combined with tissue engineering for the treatment of peripheral nerve injury, have recently been reviewed by Wei et al. [5]. More specifically, the presence of Schwann cells or similar alternatives in nerve repair constructs, such as EngNT, have improved outcome measures in peripheral nerve injury models [6,7,8,9,10]. While primary Schwann cells might be considered in nerve tissue engineering, they are not necessarily appropriate for therapeutic development due to limitations in number, invasive harvesting procedures and extensive purification requirements [11,12]. Consequently, an alternative, readily available source of therapeutic cells must be found, with the potential to generate cell populations that are safe and efficacious at a scale and cost suitable for clinical translation.

Schwann cells can be derived from human induced pluripotent stem cells (hiPSCs) via a precursor stage (Schwann cell precursors, SCPs) [13]. In addition to mature Schwann cells, these SCPs may themselves be an advantageous cell type for nerve tissue engineering. Cells at an earlier stage of differentiation exhibit a distinct molecular profile, may be easier to expand in vitro and could support regeneration to a greater degree than transplantation of terminally differentiated Schwann cells [14,15,16]. Previous studies have shown that hiPSC-derived SCPs can be stably cultured for at least 35 passages, retain their ability to differentiate into Schwann cells, and can improve regeneration in a mouse nerve injury model [13,17]. Therefore, there may be other advantages to using SCPs over mature Schwann cells in nerve tissue engineering, beyond the shorter, and therefore cheaper, manufacturing process.

**Figure 1:**
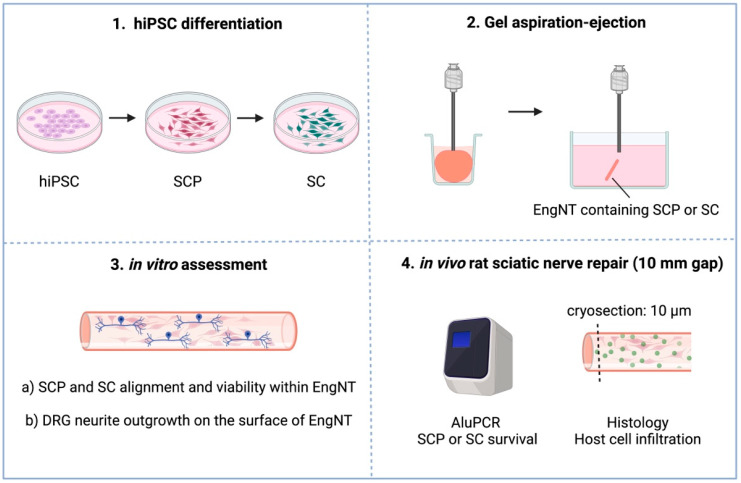
Schematic of the aims and objectives. The first objective was to differentiate human induced pluripotent stem cells (hiPSCs) into Schwann cell precursors (SCPs) and then Schwann cells (SCs), both of which would be explored as therapeutic cell types. The second objective was to form engineered neural tissue (EngNT) containing algined SCPs or SCs using the gel aspiration–ejection technique. The third objective was to conduct in vitro studies with the EngNT by (a) measuring the alignment and viability of the cells within the EngNT contruct and (b) measuring dorsal root ganglion (DRG) neurite outgrowth on the surface. The fourth objective was to implant the EngNT, containing either SCPs or SCs, into a 10 mm rat sciatic nerve gap model. After two weeks, therapeutic cell viability was assessed using AluPCR and host cell infiltration was measured histologically.

In recent years, a simplified technique for the scalable production of EngNT has been developed, called gel aspiration–ejection, where a low-density, hyper-hydrated collagen hydrogel is drawn into a cannula under negative pressure, simultaneously stabilising and aligning the collagen matrix and embedded cells [18]. This circumvents previous requirements for cell-mediated contraction and alignment [6], enabling a broad range of different cell types and cell seeding densities to be considered. In this work, we aimed to compare the survival and therapeutic potential of hiPSC-derived Schwann cells and Schwann cell precursors within EngNT in vitro and in an in vivo rodent model of peripheral nerve injury (Figure 1). To do this, Schwann cells and SCPs were differentiated from a source of hiPSCs for which Good Manufacturing Practice (GMP) equivalents are available. The molecular profile of the cells was characterised in vitro (immunocytochemistry and RT-qPCR), and then they were incorporated into EngNT using gel aspiration–ejection. Dorsal root ganglion cells were seeded on the surface of the constructs in vitro and the resulting alignment of neurites was assessed. Finally, the constructs were tested in a 10 mm rat sciatic nerve gap model to assess SCP and Schwann cell survival within the constructs after two weeks, and histology was performed to investigate infiltration of host neurons, Schwann cells and endothelial cells within the construct.

## 2. Materials and Methods

### 2.1. Culture of Human iPSC (hiPSCs) and Differentiation into SCPs and Schwann Cells

CTX-hiPSCs were obtained from ReNeuron Ltd., Bridgend, UK, and grown on tissue culture plastic coated with vitronectin (1:100 at 0.5 μg/cm^2^) in mTesR™ Plus cGMP pluripotent stem cell maintenance media in a humidified 5% CO_2_, 37 °C incubator. hiPSCs were differentiated into human Schwann cell precursors (SCPs and human Schwann cells using a protocol adapted from Kim et al. [13]. hiPSCs were detached using EDTA (0.5 mM, 1 mL/10 cm^2^) and seeded into 6-well plates coated with Geltrex (1:100 at 0.8 mL/10 cm^2^). hiPSCs were incubated for 24 h in Essential 8 Flex media before changing the media to neural differentiation media (NDM), with the media components described in Table 1.

After 6 days, human neuregulin−1 (50 ng/mL) was added to the NDM to make Schwann cell precursor differentiation media (SCPDM). SCPDM was changed daily, and differentiating cells were passaged on reaching a confluency of more than 90%. After 18 days in SCPDM, SCPs were maintained in SCPDM containing neuregulin−1 (100 ng/mL) (Schwann cell precursor maintenance media). SCPs were replated at a density of 14,000/cm^2^ in Schwann cell differentiation media (SCDM) to differentiate SCPs into Schwann cells. After 3 days, media was changed to SCDM without forskolin or retinoic acid and after a further 2 days, PDGF-BB was also withdrawn. SCPs were maintained in Schwann cell maintenance media until required for downstream applications or cryopreserved.

### 2.2. Primary Dorsal Root Ganglia Neurons

Dorsal root ganglia (DRGs) were dissected from adult female Sprague Dawley rats, cleaned to remove nerve roots and connective tissue, incubated in collagenase (0.125%) for 90 min at 37 °C and then dissociated by trituration. Cell suspensions were washed in high glucose DMEM + 10% FBS + 1% Penicillin/Streptomycin (100 U/mL and 100 µg/mL respectively) with centrifugation at 200× g for 5 min. Cells were then cultured in poly-D-lysine coated flasks in the presence of cytosine arabinoside (Ara-C) at 10 mM for 24 h.

### 2.3. Tissue Engineering

A volume of 80% Type I rat tail collagen (First Link (UK) Ltd., Wolverhampton, UK, 2 mg/mL in 0.6% acetic acid) was mixed with 10% volume of 10× minimum essential media (MEM) and neutralised with sodium hydroxide before adding 10% volume of SCPs or Schwann cells in their respective maintenance media (1 million cells/mL). This mixture was cast within a 48-well plate (1 mL per well) and left to set for 30 min at 37 °C. Acellular gels were produced using the same protocol using 10% volume cell culture media, minus the cells.

A flat-ended 16G cannula attached to an angioplasty inflation device was inserted into the collagen gel and the piston was withdrawn to aspirate the gel into the cannula [18]. Once the gel was in the cannula, the aspiration process was stopped, and positive pressure was applied to eject the EngNT construct controllably. Viability was determined using ReadyProbes™ Cell Viability Imaging Kit, Blue/Red and cell alignment analysis was performed using CellMask™, to stain the cell plasma membrane. To measure neurite outgrowth, DRGs were seeded onto the surface of acellular, SCP and Schwann cell EngNT constructs and incubated for 60 h before fixing with 4% paraformaldehyde.

### 2.4. Immunocytochemistry

Cells on coverslips were washed with PBS and fixed for 15 min with 4% paraformaldehyde. Coverslips were washed and then permeabilised with 0.5% Triton-X for 15 min at room temperature; then they were blocked with 5% serum with 0.5% Triton-X for 15 min at room temperature. Coverslips were incubated at 4 °C overnight with the primary antibody (Table 2), washed in PBS and incubated with the complementary secondary antibody (Table 2) for 45 min at room temperature. Coverslips were finally incubated with DAPI (1:1000 dilution) for 10 min at room temperature, washed in PBS and mounted with VectaShield Vibrance (Vector Laboratories Inc., Newark CA, USA). For immunocytochemistry of 3D constructs, primary antibodies were incubated for 3 h at room temperature and the secondary antibodies for 90–120 min at room temperature. Omission of primary antibodies was used as a control.

### 2.5. Imaging and Image Analysis

Three pre-determined fields from each coverslip were imaged using a Zeiss AxioLab A1 fluorescence microscope and immunopositive cells were determined as a percentage of the total number of DAPI-stained cells.

Neurite growth and cell viability within 3D constructs cultured in vitro were imaged using a confocal microscope (Zeiss LSM710). Neurite length and orientation were measured using Volocity™ software v6.5.1 (Perkin Elmer), comparing the angle of each neurite to the long axis of the construct. All neurites within the construct were measured.

### 2.6. RNA Extraction, Retro-Transcription, and RT-qPCR

RNeasy Plus Mini Kit (Qiagen) was used to extract RNA from samples according to the manufacturer’s instructions. cDNA was synthesised using a GoScript Reverse Transcriptase kit (Promega) and a SimpliAmp Thermal Cycler (Applied Biosystems). RT-qPCR was performed with a QuantStudio3 instrument (Applied Biosystems) and analysed with QuantStudio Design and Analysis Software v1.5.1 (Applied Biosystems). PCR reactions were run in duplicate using the Power SYBR Green PCR Master Mix (Thermo, ref: 4368708). The amplification products were analysed by performing a melting curve at the end of the PCR. Data were normalised to the mRNA expression of three reference genes: hypoxanthine guanine phosphoribosyl transferase (HPRT1), ribosomal protein S18 (RPS18), and TATA-box binding protein (TBP). Primer sequences for RT-qPCR are listed in Table 3. A fold change of greater than ±3 was considered to be of interest when comparing differentiated cells to hiPSCs and statistical tests were performed where differences exceeded this threshold.

### 2.7. Rat Sciatic Nerve Repair

All surgical procedures were performed in accordance with the UK Animals (Scientific Procedures) Act (1986) and the European Communities Council Directives (86/609/EEC) and approved by the UCL Animal Welfare and Ethics Review Board (AWERB). Adult male Wistar rats (250–300 g) (Charles River, Wilmington, MA, USA) were housed in a controlled environment (12 h day–light cycle, controlled temperature, and humidity) with free access to food and water. Upon arrival, they were randomised into groups and acclimated for a week. Rats were given daily injections of the immunosuppressant cyclosporin A (Sandimmune, Novartis, at 10 mg/kg) to prevent immune rejection of the xenograft. Anaesthesia was performed using isoflurane (5% for induction and 3% for maintenance) and the animals were monitored throughout the procedure to ensure depth of anaesthesia. Under an operating microscope, the sciatic nerve was released from the surrounding tissue and transected to create a gap of 10 mm. EngNT constructs were trimmed to be 1 cm long, implanted within a silicone tube and sutured to the sciatic nerve stumps using 10–0 sutures. Fourteen days after the surgery, rats were culled and tissue was harvested and the proximal half was fixed in PFA 4%.

### 2.8. Alu PCR

The distal half of the EngNT was used for Alu PCR to quantify the number of human cells present in the construct 14 days after transplantation. DNA was extracted using an AllPrep DNA/RNA kit (Qiagen) according to the manufacturer’s instructions. Alu PCR was performed using published primers and a published probe (Table 4), designed to be specific for human cell quantification among rodent cells [19], and using TaqMan Universal Master Mix II (Applied Biosystems). Two calibration curves were run using SCPs or Schwann cells at six different concentrations. Alu PCR was performed with a QuantStudio3 instrument (Applied Biosystems) and analysed with QuantStudio Design and Analysis Software v1.5.1 (Applied Biosystems). Alu PCR reactions were run in duplicate.

### 2.9. Histology

The proximal half of the EngNT constructs were used for immunofluorescence measurements. Cryosections (10 μm) were cut with a cryostat (Thermo Scientific, HM525) 0.2 mm from the proximal end and mounted onto a glass slide. Cyrosections were blocked using 5% normal goat serum and 0.5% Triton-X 100 in PBS for 1 h at room temperature. Primary antibodies (Table 5) were incubated for 24 h at 4 °C and then slides were washed then incubated for 24 h at 4 °C with the relevant secondary antibodies (1:300) and Hoechst 33,342 (Thermo Scientific, H3570) (1:1000). Slides were scanned on a NanoZoomer S60 Digital slide scanner (Hamamatsu, Japan). Schwann Cells (S100) and endothelial cells (CD31) were measured using Volocity™ software v6.5.1 (PerkinElmer) and axons (neurofilament) were counted using a fluorescence microscope (Zeiss AxioLab A1). Samples were excluded from histological analysis if sections could not be obtained or if they were outliers, based on the Grubbs outlier test.

## 3. Results

**Figure 2:**
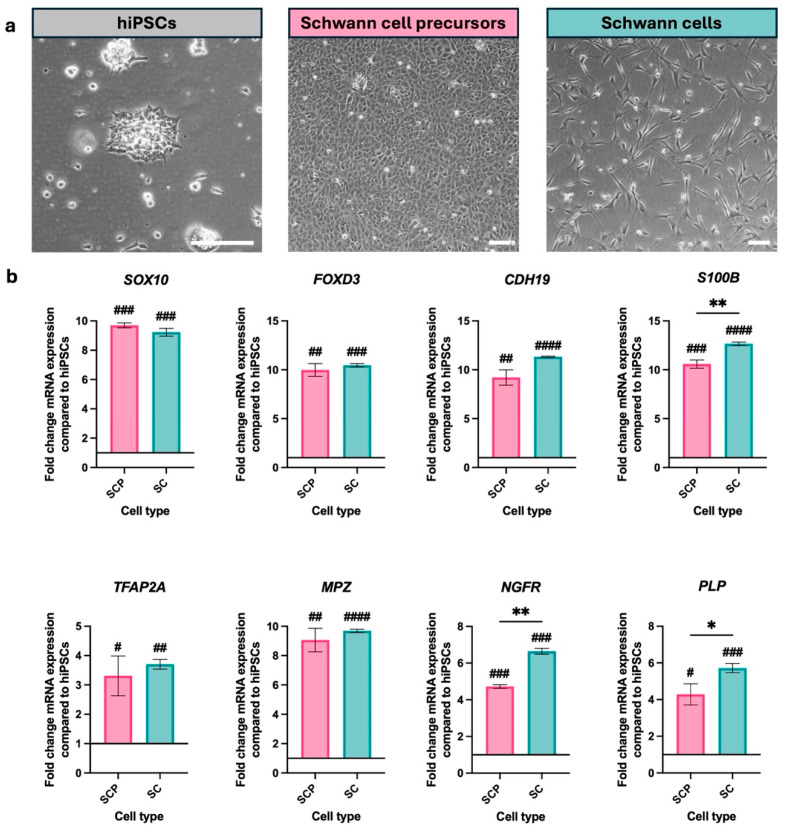
Characterisation of human induced pluripotent stem cells (hiPSCs), Schwann cell precursors (SCP) and Schwann cells (SC). (**a**) Phase contrast micrographs showing differentiation from hiPSCs, to Schwann cell precursors (24 days after initiation of differentiation) and then to Schwann cells (31 days after initiation of differentiation). Scale bars 100 µm. (**b**) RT-qPCR for the differentiation of hiPSCs to Schwann cells via SCPs. Data are mean ± SD from 3 independent differentiation inductions, each of which yielded SCPs at 24 days and Schwann cells at 31 days. A one-sample t-test was performed to compare SCPs and Schwann cells (SCs) to the reference hiPSC population, which was given a hypothetical value of 1, where # *p* <  0.05, ## *p* < 0.01, ### *p* < 0.001, #### *p* < 0.0001. A paired t-test was performed to compare the SCPs and Schwann cells where * *p* <  0.05, ** *p* <  0.01.

This is the first time that differentiation of the CTX-hiPSC line into SCPs and Schwann cells has been reported. The differentiation was conducted using the protocol developed by Kim et al. [13], with the modifications of Geltrex-coated flasks and a higher seeding density of 14,000 cells/cm^2^, as described in [17]. Differentiation from hiPSCs to SCPs took 24 days, with a further 7 days required to differentiate SCPs to Schwann cells (31 days total). These Schwann cells, produced on day 31, were maintained for up to 7 days in the Schwann cell maintenance media before being used in experiments. During differentiation, morphological changes were observed. From day 0 to day 24, hiPSCs transitioned from colonies of cells with a typical undifferentiated morphology to densely packed monolayers with a uniform shape. From day 24 to day 31, cells become bipolar with two extensions, sometimes appearing to form connections with those of other cells (Figure 2a).

The cells were characterised at their developmental stages using RT-qPCR, confirming the distinct molecular profiles associated with SCPs and Schwann cells. The expression of genes associated with Schwann cell differentiation were significantly upregulated in SCPs and Schwann cells compared with the starting hiPSC population (Figure 2b, Table S1). Differences in expression of these genes were compared between SCPs and Schwann cells. The neural crest and SCP-associated genes *SOX10, FOXD3, CDH19* and *TFAP2a* were expressed similarly by both SCPs and Schwann cells. Markers associated with Schwann cell maturation showed some differences, with *S100B, NGFR*, and *PLP* expressed at a significantly higher level in Schwann cells compared with SCPs. *MPZ* which is associated with myelination showed similar levels of expression in the SCPs and Schwann cells. As this was the first time this differentiation protocol was used with the CTX-hiPSCs, immunofluorescence was used to characterise the SCP and Schwann cells (Figure S1). A decrease in Oct4 positive cells and an increase Sox10 and p75^NTR^ positive cells was observed as differentiation progressed from hiPSCs to SCPs. Only the differentiated Schwann cells were immunopositive for S100b.

**Figure 3:**
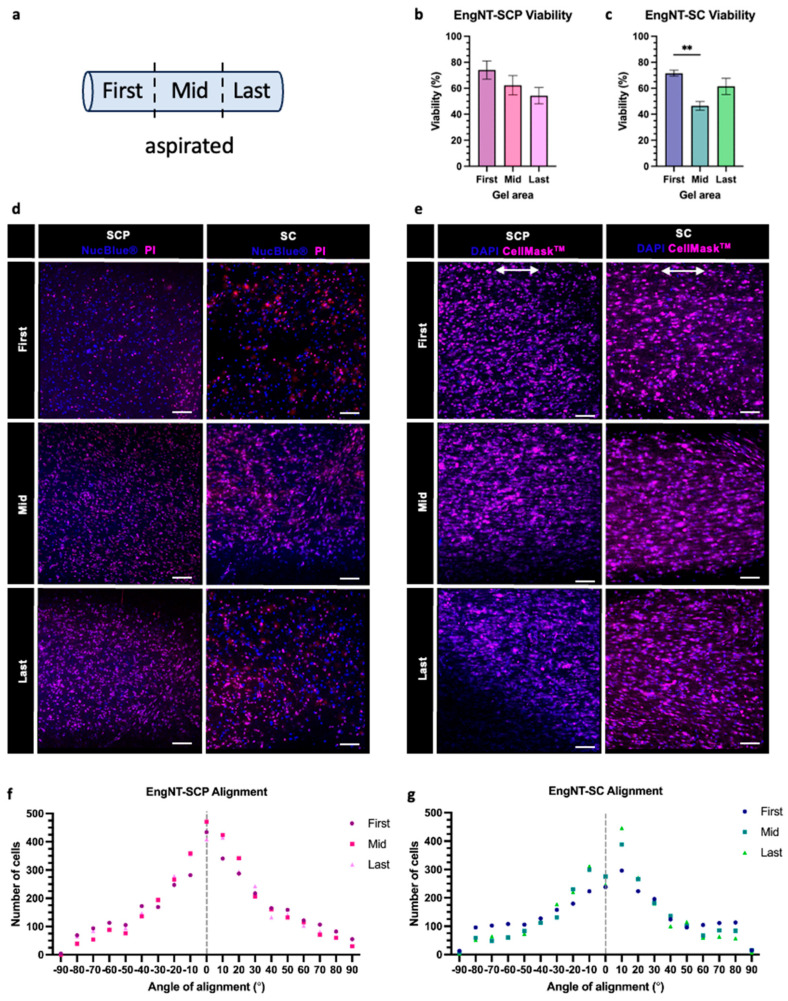
Viability and alignment of hiPSC-derived Schwann cell precursors (SCPs) and Schwann cells (SCs) in EngNT constructs. (**a**) Three regions within each construct were defined, based on which part of the EngNT was aspirated first into the cannula. (**b**,**c**) Percentage viability of SCP and SC was measured within EngNT after 24 h in vitro (mean ± SEM) using a one-way ANOVA with Tukey’s multiple comparison test, ** *p* < 0.0021. n = 6 EngNT constructs from 3 differentiation inductions. (**d**) Representative images used to quantify the viability of the SCP (**left**) and SC (**right**) in the first (**top**), middle (**middle**) and last (**bottom**) aspirated EngNT regions using NucBlue^®^ to identify all cells (blue) and propidium iodide (PI) to identify dead cells (magenta), arrow indicates the longitudinal axis of EngNT. (**e**) Representative images used to quantify the alignment of SCPs (**left**) and SCs (**right**) stained with CellMask™ (magenta) to visualise the plasma membrane and DAPI (blue) to visualise cell nuclei. Histograms of SCP (**f**) and SC (**g**) alignment within EngNT. Dotted line signifies alignment parallel to EngNT longitudinal axis.

Once differentiated and characterised, SCPs or Schwann cells were mixed with collagen to form cellular hydrogels, which were then stabilised and aligned via gel aspiration and ejection to make EngNT (18). Cell survival and alignment were confirmed after 24 h in culture, with mean cell viability 64 ± 10% in the SCP EngNT construct and 60 ± 13% in the Schwann cell EngNT construct. Regional variability was explored by defining three distinct sections of the EngNT constructs: the first, middle, and last thirds, in order of aspiration (Figure 3a, Table S2). No significant differences were observed in cell viability between areas of the SCP construct (Figure 3b), whereas the Schwann cell construct exhibited some regional variability (Figure 3c). Viability and alignment were measured using image analysis of confocal z-stacks that captured the full depth of the constructs. Extended focus views of representative images indicate the dense cellular structure throughout the engineered tissue (Figure 3d,e). SCPs and Schwann cells were highly aligned parallel to the longitudinal axis throughout the EngNT constructs (Figure 3f,g).

**Figure 4:**
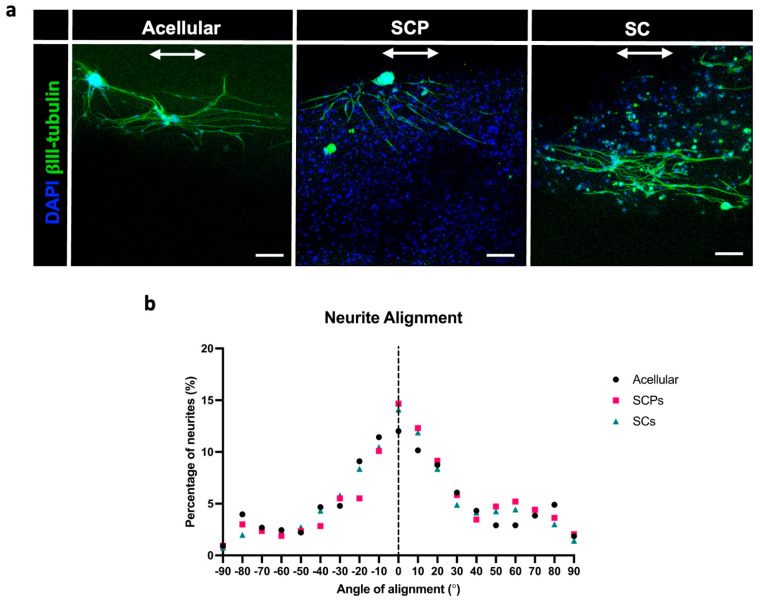
EngNT constructs supported aligned neurite growth in vitro. (**a**) Representative images showing neurite outgrowth of DRG neurons on acellular (**left**), Schwann cell precursor (SCP; **middle**) and Schwann cell (SC; **right**) EngNT constructs. Neurons stained with β-III-tubulin (green) and DAPI nuclei stain (blue). Scale bar 50 µm, arrow indicates the longitudinal axis of EngNT. (**b**) Histogram of neurite alignment to EngNT longitudinal axis. The dotted line signifies alignment parallel to the EngNT longitudinal axis (n = 9 EngNT constructs from 3 differentiation inductions). There were differences in the absolute number of neurons that grew in co-culture with the EngNT constructs; therefore, to allow direct comparison of the distribution of alignment between the groups, the number of neurites in each condition were expressed as a percentage. Raw data available (Table S3).

To explore the ability of the EngNT to support neurite outgrowth in vitro, primary sensory neurons from rat DRGs were co-cultured on the surface of acellular, SCP and Schwann cell EngNT for 60 h. Both acellular and cellular EngNT containing SCP or Schwann cells supported and guided neurite growth in an aligned manner with approximately 60% of neurites growing within ± 30° of the long axis of the EngNT (Figure 4).

**Figure 5:**
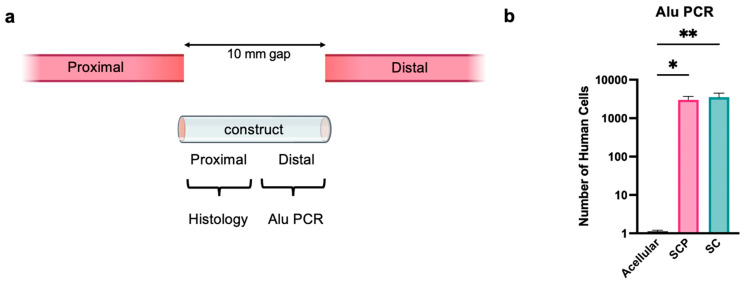
Differentiated Schwann Cells and their precursors were used to form EngNT and survive after two weeks in vivo. (**a**) Acellular, Schwann cell precursor (SCP) and Schwann cell EngNT constructs were used to repair a 10 mm gap in a transected rat sciatic nerve and then harvested after 2 weeks. (**b**) Alu PCR indicates the number of human cells remaining in half of the construct after two weeks in vivo (n = 6, mean ± SD). One sample was excluded from both the acellular and SCP groups (n = 5) based on the Grubbs outlier test. One-way ANOVA (Kruskal–Wallis test) with Dunn’s multiple comparison test was used where * *p* < 0.05, ** *p* < 0.01.

Having established that both SCPs and Schwann cells differentiated from hiPSCs could be used to form EngNT constructs that supported aligned neurite growth in vitro, an initial in vivo test was performed to assess their ability to survive, integrate and support cellular infiltration, including host neurons, Schwann cells and endothelial cells. EngNT constructs (made using SCPs, Schwann cells or acellular controls) were used to bridge a 10 mm rat sciatic nerve gap and after 2 weeks, the tissue was analysed. The distal half of the EngNT was used to quantify the remaining number of SCPs or Schwann cells and transverse sections were taken from the proximal end of the EngNT to investigate cellular infiltration (Figure 5a). Alu qPCR was used to quantify the number of surviving transplanted cells and significantly more human cells were detected after 2 weeks in the SCP and Schwann cell groups than in the baseline acellular group (Figure 5b). The mean number of cells surviving in half of the SCP construct was 6078 *±* 3132 compared with 3508 *±* 970 in the Schwann cell construct. This represents approximately 1% of the original transplanted cell population, with no significant difference between the two cellular EngNT groups.

**Figure 6:**
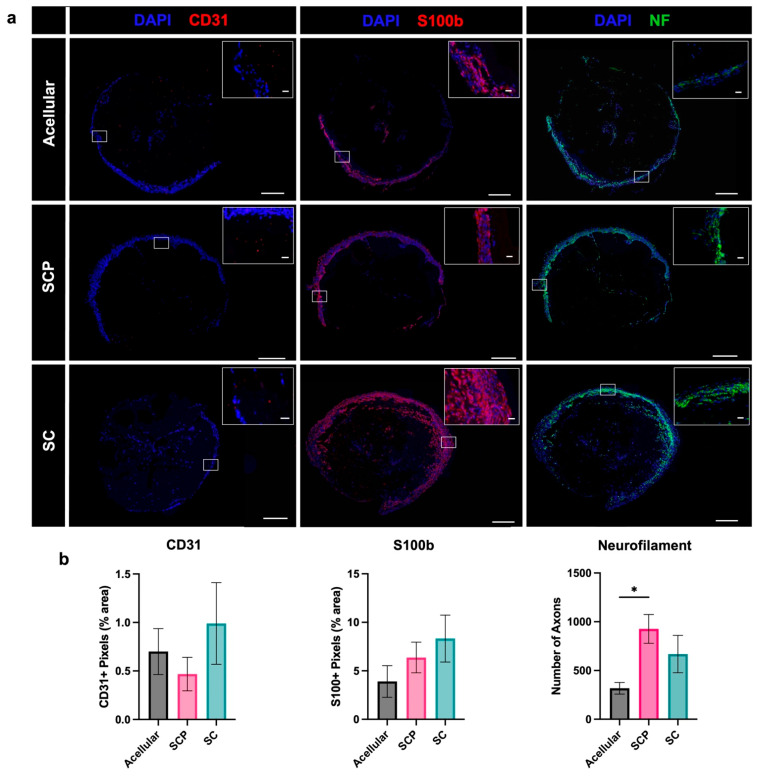
Histological characterisation of endogenous cell infiltration within EngNT after two weeks in vivo. (**a**) Infiltrating cells were detected using immunofluorescence in transverse sections through the proximal end of the constructs. (**b**) This enabled quantification of endothelial cell CD31 (red) and Schwann cell (S100b) (red) area, and the number of neurofilament (green) positive neurons that had entered the EngNT (mean ± SEM). Samples analysed per condition varied (N = 4–6) as some tissue sections were of unreliable quality and Grubbs’ Test excluded extreme outliers (*p* < 0.05). Scale bar 100 µm, scale bar in inset 20 µm. One-way ANOVA with Tukey’s multiple comparison test * *p* < 0.05.

Transverse sections from the proximal end of the EngNT showed the presence of endothelial cells, Schwann cells and neurites in a circular pattern within the outer part of the grafts (Figure 6a). Quantification of this cellular ingrowth indicated no significant differences between groups for endothelial cells (CD31) and Schwann cells (S100b), but there was a significant (*p* = 0.04) 3-fold increase in the number of infiltrating axons (neurofilament) detected in the EngNT-SCP group compared with the acellular control (Figure 6b). The mean number of axons in the EngNT-Schwann cell group was approximately double that of the acellular control, although this difference was not statistically significant (*p* = 0.32).

## 4. Discussion

Stem cell-derived therapeutic cells have shown promise in treating nerve injury in a wide range of preclinical models and some clinical trials, where they mimic the support and guidance provided by Schwann cells in an autograft. Sources of stem cells that have been explored include tissue-derived multipotent stem cells and embryonic stem cells, with iPSCs offering advantages in terms of quality control, ethics and supply. Recent preclinical and clinical studies reporting stem cell therapies for nerve regeneration have been reviewed by Wynne et al. [20].

In this study, SCPs and Schwann cells were successfully differentiated from hiPSCs, with changes in morphology, and molecular expression profile at the gene and protein level consistent with those seen in previous similar studies [12,13,17]. There were differences in the expression of characteristic markers between the SCPs and the Schwann cells, in particular, increased *P75NTR*, *S100B* and *PLP*, which corresponded to the changes associated with differentiation from SCP to Schwann cells [21].

Both the SCP and Schwann cell populations were amenable to being incorporated into EngNT using the rapid and scalable tissue engineering technique of gel aspiration–ejection [22,23,24]. This approach has been used previously to make EngNT containing rat Schwann cells [18] and human umbilical vein endothelial cells [25], but this is the first report of the technique being used with hiPSC-derived cells. There was a reasonable level of cell viability throughout the constructs, with no noticeable differences in overall viability between the SCPs and the Schwann cells. In future studies, it would be interesting to determine how the molecular profile of the SCPs and Schwann cells changes, following incorporation into EngNT. Future refinements to the gel aspiration–ejection technique such as automation and the use of different cannula sizes may further improve the viability of the incorporated cells, as well as improve the scalability of the production technology [25]. In addition to viable support cells, EngNT needs to provide an aligned architecture to support and guide regenerating neurites [2]. Both the SCPs and Schwann cells were highly aligned parallel to the longitudinal axis throughout the EngNT constructs. Overall, these results showed that both hiPSC-derived populations tested here could be incorporated into EngNT, with SCPs and Schwann cells exhibiting similar viability and alignment in the resulting stabilised collagen constructs. The successful generation of engineered tissues enabled both cell populations to be taken forward for further testing in vitro and in vivo.

Co-culture with rat DRG-derived neurons was used as an in vitro model to test the ability of the EngNT constructs to support neuronal regeneration [26]. The neurons survived and extended neurites in a highly directional manner, indicating the ability of the EngNT samples to support and guide regeneration. The alignment conferred on the neurites in vitro by the EngNT constructs was similar to that reported previously with EngNT made using other types of cells [6,18,25]. No difference in the alignment of neurite growth was identified in the co-culture assay between EngNT containing the different cell types or the acellular constructs. The ability of the acellular constructs to guide neurite growth to the same extent as the cellular EngNT indicates the contribution that collagen fibril alignment makes in these engineered tissues [23].

A rat sciatic nerve injury model was used to explore how the EngNT constructs behave after transplantation. After two weeks the repairs were analysed in terms of cell infiltration and transplanted cell survival. Alu PCR was used to specifically detect human DNA still present in the EngNT implanted in rats [19]. Human cells were still detectable in both the SCP and Schwann cell groups, with no significant difference between them. The number of cells remaining at this time point was relatively low in comparison to the number that were transplanted. The number of surviving cells required to support regeneration is currently unknown. It would be interesting to investigate this further, for example by measuring the number of viable Schwann cells in an autograft under similar conditions. The infiltration of endothelial cells (CD31) and Schwann cells (S100b) was similar in all groups and cross-sectional micrographs indicated this was localised predominantly to the outer region of the constructs. The growth of neurites (neurofilament) within the repairs also corresponded to this location, which is similar to observations made previously in EngNT graft repairs [27]. There was a trend towards more neurites in the cellular EngNT groups compared with the acellular group, with the SCP EngNT constructs supporting a significant ~3-fold greater number of neurites than the acellular constructs. In this preliminary in vivo study, cell survival and host cell infiltration were only assessed at a two-week time point, meaning it was not appropriate to measure functional or behavioural outcomes. A longer time point (for example 8 weeks) would be required in order to measure such outcomes and determine whether the inclusion of SCPs or SCs resulted in a greater therapeutic effect than an acellular construct. Future work could explore longer-term effects and longer nerve gaps, enabling comprehensive functional assessment to be conducted including electrophysiology and sciatic function index [13]. Transmission electron microscopy could also be used to investigate the regeneration of structures such as myelin and extracellular matrix architecture. Furthermore, it would be beneficial to use group sizes that would improve the statistical power and enable robust comparisons to be drawn between groups in models with longer gaps and durations.

This initial investigation of SCPs and Schwann cells derived from hiPSCs in EngNT indicates that both cell populations can potentially be useful in nerve tissue engineering. The feasibility of differentiating hiPSCs to yield both cell types has been demonstrated, and they can each be used to form EngNT and support cell infiltration and neurite growth in vivo. There was no clear advantage to using the fully differentiated Schwann cells rather than the SCPs based on any of the in vitro or in vivo outcome measures. However, SCPs demonstrated their capacity to promote infiltrating endogenous axons in the in vivo experiment. This may indicate that SCPs are a better option than Schwann cells as a therapeutic cell type in nerve tissue engineering. Transplantation of SCPs has previously been proposed to be potentially useful therapeutically in peripheral nerve and central nervous system applications [13,28,29]. These results add further support to that approach, indicating that SCPs derived from hiPSCs may have advantages over Schwann cells in nerve tissue engineering. In addition, there are other advantages in terms of logistics for the use of SCPs rather than Schwann cells. Not only is the differentiation time shorter and more efficient, but SCPs can also readily proliferate, and populations can be expanded and stored more easily than their fully differentiated Schwann cell counterparts.

## 5. Conclusions

This study has successfully differentiated and characterised SCPs and Schwann cells from the CTX-hiPSC line for the first time. It demonstrated an effective method for incorporating each cell type into EngNT and conducted initial tests in an animal model of nerve injury, demonstrating the cells can survive after two weeks and promote endogenous axon infiltration. This will help to inform future developments of nerve tissue engineering approaches using hiPSC-derived SCPs in particular, since this may be a promising cell type for therapeutic use.

## Figures and Tables

**Table 1:** Media components in neural differentiation, Schwann cell precursor and Schwann cell differentiation media added, as described in Kim et al. [13].

Neural Differentiation Media (NDM)	Schwann Cell Precursor Differentiation Media (SCPDM)	Schwann Cell Differentiation Media (SCDM)	Schwann Cell Precursor Maintenance Media	Schwann Cell Maintenance Media
Advanced DMEM/F12 and Neurobasal media (1:1)	Advanced DMEM/F12 and Neurobasal media (1:1)	DMEM low-glucose	Advanced DMEM/F12 and Neurobasal media (1:1)	DMEM low-glucose
BSA (0.005% *v/v*)	BSA (0.005% *v/v*)	Retinoic acid (100 nM)	BSA (0.005% *v/v*)	Neuregulin−1 (200 ng/mL)
β-mercaptoethanol (0.11 mM)	β-mercaptoethanol (0.11 mM)	Forskolin (4 μM)	β-mercaptoethanol (0.11 mM)	FBS (1% *v/v*)
N2 supplement	N2 supplement	PDGF-BB (10 ng/mL)	N2 supplement	
B27 supplement	B27 supplement	Neuregulin−1 (200 ng/mL)	B27 supplement	
SB431542 (20 mM)	SB431542 (20 mM)	FBS (1% *v/v*)	SB431542 (20 mM)	
CT99021 (3 mM)	CT99021 (3 mM)		CT99021 (3 mM)	
GlutaMAX (2 mM)	Neuregulin−1 (50 ng/mL)		Neuregulin−1 (100 ng/mL)	

**Table 2:** Primary and secondary antibodies used for immunocytochemistry.

Primary Antibodies	Dilution	Reference
Sox10	1:40	SantaCruz, SC−365692
p75^NTR^	1:300	Cell Signalling Technologies, 8238
S100b	1:1	Dako, GA50461−2
Oct3/4	1:200	SantaCruz, SC−8629
Sox2	1:200	Millipore, MAB4343
c-Jun	1:200	Cell Signalling Technologies, 9165
Ki67	1:200	Abcam, ab15580
**Secondary Antibodies**	**Dilution**	**Reference**
Horse anti-mouse 549	1:200	Vector labs, DI−2549
Horse anti-mouse 488	1:200	Vector labs, DI−2488
Goat anti-rabbit 549	1:200	Vector labs, DI−1549
Goat anti-rabbit 488	1:200	Vector labs, DI−1488
Horse anti-goat 594	1:200	Vector labs, DI−3094

**Table 3:** Forward and reverse primers used to characterise differentiated Schwann cells and SCPs. Primers were designed using IDT PrimerQuest Tool with specificity checked using NCBI primer BLAST. Primer quality was checked using IDT UNAFold tool (secondary structure characterisation) and Beacon Designer (thermodynamic assessment).

Gene	Alias	Name	Forward Primer (5′−3′)	Reverse Primer (5′−3′)
*SOX10*		SRY-box transcription factor 10	TACACCGACCAGCCATC	GGTCAGAGTAGTCAAACTGG
*FOXD3*		forkhead box D3	GCTCATCAAGTCCGAGCCAAG	CCACCTATGATGTTCTCGATGCTG
*CDH19*		cadherin 19	CTTGTCTTGGAGCAACAG	ATCTTAGCTGGCCGATG
*NGFR*	*p75NTR*	nerve growth factor receptor	GTGAGTGCTGCAAAG	AACGTCACGCTGTC
*TFAP2A*	*AP−2*	transcription factor AP−2 alpha	GAAGCTGTCCACCTAGC	CTTTGGCAGGAAATTCGG
*MPZ*		myelin protein zero	CTACATTGACGAGGTG	AGTCTAGGTTGTGTATG
*S100B*		S100 calcium binding protein B	CTCATCAACAATGAGCTTTC	TCACATTCGCCGTCTC
*PLP1*	*PLP*	proteolipid protein 1	ACCTGCCAGTCTATTG	TGGGAGAACACCATAC
*HPRT1*		hypoxanthine phosphoribosyltransferase 1	AGGGTGTTTATTCCTCATGGAC	CCCATCTCCTTCATCACATCTC
*RPS18*		ribosomal protein S18	CAAGAGGGCGGGAGAAC	CGTGGATTCTGCATAATGGTG
*TBP*		TATA-box binding protein	ACTTCGTGCCCGAAACG	GTGGTTCGTGGCTCTCTTATC

**Table 4:** Alu PCR primer and probe sequences. The probe was modified at the 5′ end with 6-carboxy-fluorescein (6-FAM) and at the 3′ end with Black Hole Quencher 1 (BHQ1).

Primer or Probe	Sequence
Forward primer (5′−3′)	GGTGAAACCCCGTCTCTACT
Reverse primer (5′−3′)	GGTTCAAGCGATTCTCCTGC
Probe (5′−3′)	6-FAM/CGCCCGGCTAATTTTTGTAT/BHQ1

**Table 5:** Primary and secondary antibodies used in histology.

Primary Antibodies	Dilution	Reference
Neurofilament	1:300	Biolegend, 835604
CD31	1:200	Invitrogen, PA5−32321
S100b	1:200	Dako, IR504
**Secondary Antibodies**	**Dilution**	**Reference**
Goat anti-rabbit 647	1:300	Alexa Fluor, A32733
Goat anti-rabbit 555	1:300	Alexa Fluor, A32732
Goat anti-mouse 488	1:300	Alexa Fluor, A32723

## Data Availability

The original contributions presented in this study are included in the article with the SI. Further inquiries can be directed to the corresponding authors.

## Supplementary Materials

The following supporting information can be downloaded at: https://www.mdpi.com/article/10.3390/bioengineering12090904/s1, Figure S1 shows immunofluorescence characterisation of hiPSCs, SCPs and Schwann cells. Table S1 shows RT-qPCR data for the differentiation of hiPSCs. Table S2 shows data for the viability of hiPSC-derived Schwann cell precursors SCPs and Schwann cells (SCs) in EngNT constructs. Table S3 shows raw data for neurite alignment.
