# Supplementary material for: Engineered Neural Tissue (EngNT) Containing Human iPSC-Derived Schwann Cell Precursors Promotes Axon Growth in a Rat Model of Peripheral Nerve Injury
Source: Bioengineering (Basel). 2025 Aug 23;12(9):904. doi: 10.3390/bioengineering12090904 (PMC12468002; doi:10.3390/bioengineering12090904)
Supplement: Supplementary file 1 [file bioengineering-12-00904-s001.zip › bioengineering-3776567-supplementary.pdf]

Supplementary Information

Engineered Neural Tissue (EngNT) Containing Human iPSC-Derived Schwann Cell Precursors Promotes Axon Growth in a Rat Model of Peripheral Nerve Injury

Rebecca A. Powell [1,†], Emily A. Atkinson [1,†], Poppy O. Smith [1], Rickie Patani [2,3], Parmjit S. Jat [4], Owein Guillemot-Legris [1,‡] and James B. Phillips [1,*,‡]

1. UCL Centre for Nerve Engineering, Department of Pharmacology, UCL School of Pharmacy,University College London, London WC1N 1AX, UK; rebecca.powell1293@gmail.com (R.A.P.);emily.atkinson@ucl.ac.uk (E.A.A.); poppy.smith.20@ucl.ac.uk (P.O.S.); o.guillemot-legris@ucl.ac.uk (O.G.-L.)
2. The Francis Crick Institute, London NW1 1AT, UK; rickie.patani@ucl.ac.uk
3. Department of Neuromuscular Diseases, Queen Square Institute of Neurology, University College London, London WC1N 3BG, UK
4. MRC Prion Unit at UCL, UCL Institute of Prion Diseases, University College London, London W1W 7FF, UK; p.jat@prion.ucl.ac.uk

* Correspondence: Correspondence: jb.phillips@ucl.ac.uk

† Co-first authors. These authors contributed equally to this work.

‡ Co-last authors.

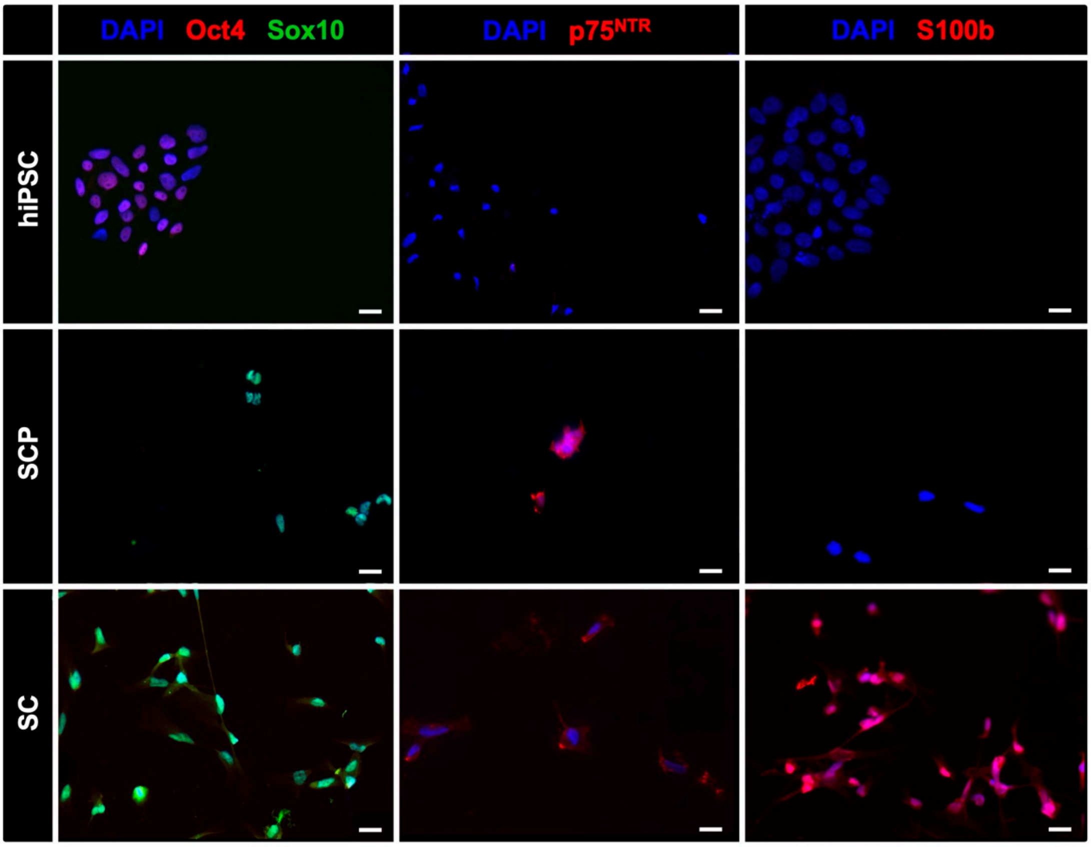

Figure S1. Immunofluorescence characterisation of hiPSCs, SCPs and Schwann cells stained with pluripotency marker Oct4 (red; left) and Schwann cell markers Sox10 (green; left), $p75^{NTR}$ (red; middle) and S100b (red; right) with DAPI (blue). Scale bar 20 µm.

Table S1. RT-qPCR for the differentiation of hiPSCs to Schwann cells via Schwann cell precursors (SCPs). Data from 3 independent differentiation inductions, each of which yielded SCPs at 24 days and Schwann cells at 31 days.

	**SCP**			**SC**			**hSCs**		
**SOX10**	9.8	9.5	9.8	8.9	9.4	9.4	6.7	6.3	7.7
**FoxD3**	10.7	9.4	9.9	10.5	10.3	10.6	7.0	6.1	7.6
**CDH19**	9.7	8.3	9.6	11.3	11.4	11.3	14.6	13.8	15.3
**S100B**	10.9	10.1	10.8	12.8	12.5	12.7	15.4	14.2	15.5
**TFAP2A**	3.7	2.5	3.7	3.6	3.9	3.6	2.4	2.1	2.3
**MPZ**	9.3	8.2	9.7	9.6	9.8	9.7	9.8	8.3	10.8
**NGFR**	4.7	4.6	4.8	6.6	6.8	6.5	5.8	3.8	5.8
**PLP**	4.0	3.9	4.9	5.7	5.5	6.0	7.2	5.4	7.6

Table S2. Viability of hiPSC-derived Schwann cell precursors SCPs and Schwann cells (SCs) in EngNT constructs. Complete data set (n=6 constructs) for cell viability within the "first", "mid" and "last" aspirates areas of each construct.

**Schwann cells (SC)**				
**Area**	**Total Cells**	**Dead Cells**	**Live Cells**	**Percentage Viability**
first	970	318	652	67.2
mid	2100	1354	746	35.5
last	1166	310	856	73.4
first	1119	329	790	70.6
mid	2094	1021	1073	51.2
last	1162	328	834	71.8
first	1067	391	676	63.4
mid	2297	1334	963	41.9
last	1176	301	875	74.4
first	1251	317	934	74.7
mid	1182	488	694	58.7
last	781	437	344	44.0
first	1213	303	910	75.0
mid	1242	630	612	49.3
last	1285	766	519	40.4
first	1427	305	1122	78.6
mid	1396	805	591	42.3
last	1355	478	877	64.7
**Schwann cell precursors (SCP)**				
**Area**	**Total Cells**	**Dead Cells**	**Live Cells**	**Percentage Viability**
first	1588	398	1190	74.9
mid	2251	856	1395	62.0
last	1474	1015	459	31.1

first	1724	519	1205	69.9
mid	2005	539	1466	73.1
last	1586	782	804	50.7
first	1229	114	1115	90.7
mid	1694	157	1537	90.7
last	1681	476	1205	71.7
first	972	94	878	90.3
mid	1615	640	975	60.4
last	1551	498	1053	67.9
first	1588	398	1190	74.9
mid	2251	856	1395	62.0
last	1474	1015	459	31.1
first	1724	519	1205	69.9
mid	2005	539	1466	73.1
last	1586	782	804	50.7

Table S3. Raw data used to generate the histogram of neurite alignment to EngNT longitudinal axis (Figure 4).

Bin	Schwann cell precursors	Schwann cells	Acellular
-90	2	9	2
-85	10	14	9
-80	6	15	20
-75	11	25	18
-70	6	27	10
-65	8	12	12
-60	3	24	9
-55	9	16	11
-50	9	29	9
-45	7	42	17
-40	14	30	17
-35	4	37	25
-30	25	58	21
-25	9	53	26
-20	17	76	40
-15	27	88	32
-10	33	89	55
-5	50	104	53
0	43	138	48
5	41	111	62
10	32	107	35
15	33	86	44

20	25	76	39
25	35	66	29
30	19	35	28
35	12	37	23
40	8	38	18
45	18	33	14
50	14	33	12
55	15	41	10
60	20	40	12
65	11	37	15
70	18	37	13
75	14	31	20
80	9	27	23
85	11	26	16
90	6	11	10
